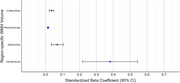# Differential Associations of Regional White Matter Hyperintensities with Hippocampal Atrophy

**DOI:** 10.1002/alz70860_106311

**Published:** 2025-12-23

**Authors:** Hitesh Pradhan, Priya Chatterjee, Jonas S Sundarakumar, CBR‐SANCOG

**Affiliations:** ^1^ Centre for Brain Research, Indian Institute of Science, Bangalore, Karnataka, India

## Abstract

**Background:**

Previous studies have reported the association of white matter hyperintensities (WMH), an indicator of cerebrovascular change, with hippocampus. And some studies have also found that hippocampal atrophy (HA) is associated with poor cognitive functions. However, the region‐specific association of WMH with HA, especially in rural Indian population, remains unclear and unexplored.

**Method:**

We analysed baseline MRI data of participants aged 45+ years (*n* = 1253) from the Centre for Brain Research – Srinivaspura Aging NeuroSenescence and COGnition (CBR‐SANSCOG) study, collected between January 2018 and September 2024. We excluded subjects with dementia assessed using Clinical Dementia Rating (CDR ≥1) and gross brain pathology on MRI. Composite cognitive score was computed by averaging normalized domain‐wise scores across visuospatial, attention, memory and language from culturally adapted computerized neurocognitive test battery (COGNITO, Computerized Assessment of Adult Information Processing). HA score was computed as the ratio of the sum of hippocampal and inferior lateral ventricle volumes to hippocampal volume, which was normalized for age and sex. Higher HA score indicates higher hippocampal atrophy. WMH volume across periventricular (pWMH), subcortical (sWMH), infratentorial (iWMH), and juxtacortical (jWMH) regions were computed using deep‐learning‐based Lesion Segmentation Toolbox. Generalized linear model (GLM) was used to investigate the association between WMH and HA, and cognitive performance, adjusting for age, sex, and education.

**Result:**

In our study (56.53±8.98 years, 35% female), we found that higher HA was associated with poorer attention (β = ‐0.33, 95% CI [‐0.07, ‐0.60], *p* = 0.01) and composite cognitive score (‐0.18, [‐0.01, ‐0.34], *p* = 0.03). Further, we found that higher pWMH (0.01, [0.01, 0.02], *p* <0.001), sWMH (0.07, [0.03, 0.10], *p* <0.001), iWMH (0.38, [0.21, 0.54], *p* <0.001), and jWMH (0.03, [0.02, 0.04], *p* <0.001) volumes were associated with higher HA.

**Conclusion:**

Our study found that region‐specific contribution of WMH volume towards hippocampus atrophy differs, wherein contribution of infratentorial WMH volume was comparatively higher than other regions. This study highlights the need of detailed assessment of occurrence of WMH in the MRI and provide cerebrovascular‐risk modifying interventions to potentially slow HA and preserve cognitive function in aging Indian population.